# Incorporating Medical Supply and Demand into the Index of Physician Maldistribution Improves the Sensitivity to Healthcare Outcomes

**DOI:** 10.3390/jcm11010155

**Published:** 2021-12-28

**Authors:** Atsushi Takayama, Hemant Poudyal

**Affiliations:** 1 Center for Innovative Research for Communities and Clinical Excellence (CiRC2LE), Fukushima Medical University, Fukushima City 960-1295, Fukushima, Japan; m05054at@live.jp; 2 Population Health and Policy Research Unit, Graduate School of Medicine, Kyoto University, Kyoto 606-8501, Kyoto, Japan

**Keywords:** death rate, healthcare disparities, health status disparities, medical resource allocation, stroke

## Abstract

Background: Since the association between disparity in physician distribution and specific healthcare outcomes is poorly documented, we aimed to clarify the association between physician maldistribution and cerebrovascular disease (CeVD), a high-priority health outcome in Japan. Methods: In this cross-sectional study, we conducted multivariable regression analysis with the Physician Uneven Distribution Index (PUDI), a recently developed and adopted policy index in Japan that uniquely incorporates the gap between medical supply and demand, as the independent variable and CeVD death rate as the dependent variable. Population density, mean annual income, and prevalence of hypertension were used as covariates. Results: The coefficient of the PUDI for the CeVD death rate was −0.34 (95%CI: −0.49–−0.19) before adjusting for covariates and was −0.19 (95%CI: −0.30–−0.07) after adjusting. The adjusted R squared of the analysis for the PUDI was 0.71 in the final model. However, the same multivariable regression model showed that the number of physicians per 100,000 people (NPPP) was not associated with the CeVD death rates before or after adjusting for the covariates. Conclusion: Incorporating the gap between the medical supply and demand in physician maldistribution indices could improve the responsiveness of the index for assessing the disparity in healthcare outcomes.

## 1. Introduction

The population of Japan is shrinking and aging at an unprecedented rate. In 2019, the Japanese population declined by −2.2%, while 28.4% of its residents were aged ≥65 years [1]. Nevertheless, several healthcare outcomes, especially those related to chronic non-communicable diseases, continue to improve. For example, death rates associated with malignant neoplasm, cardiovascular diseases, and cerebrovascular diseases (CeVD), the top three leading causes of death in 2018 [2], have been declining [3,4] with the most substantial decrease in death rates for CeVD in both men and women [4]. The death rates for CeVD (including cerebral infarction, cerebral hemorrhage, subarachnoid hemorrhage, and any other types of CeVD) decreased from 175.8 per 100,000 people in 1970 to 87.1 in 2018 [5,6].

Despite the declining death rates, mitigating the risk factors of CeVD is one of the top priorities of primary care physicians in Japan [7,8] for several reasons: First, the absolute number of patients with CeVD is expected to increase, and most of the CeVD disease burden in Japan is within the growing older population (> 60 years) [9]. Second, the resulting permanent damage after a cerebrovascular event is the leading cause of nursing care service admissions following dementia among the Japanese elderly [10]. Finally, since the policy and guidelines for the prevention, treatment and management of CeVD and associated risk factors such as hypertension and smoking are relatively well-established, the taxpayers should have access to standardized overall CeVD care under the Japanese universal health insurance policy.

However, the unequal distribution of physicians has been a central issue for healthcare delivery both in [11] and outside Japan, with several countries including China [12], India [13], England [14], Canada [15], United States [16], and across the European region [17] reporting disparities in healthcare delivery because of physician maldistribution.

In the past five decades, several policies have been implemented in Japan to address physician maldistribution. During the 1970s, 34 new medical schools were established to achieve a national physician/population ratio of 150/100,000 [18]. Although this policy effectively doubled the Number of Physicians per 100,000 People (NPPP) from 114.7 in 1970 to 258.8 in 2018 [19], the inequality in physician distribution has not improved [20,21]. Moreover, hospital-dependent specialties such as anesthesiologists and radiologists remain unevenly distributed [22]. There is also a growing urban-rural disparity in physician supply in many specialties, including internal medicine, surgery, orthopedics, and obstetrics/gynecology [11].

In 2004, the Ministry of Health, Labor, and Welfare (MHLW) implemented a mandatory “super-rotation” system where all trainees were required to rotate work in all relevant specialties over two years [23]. More recently, in 2018, the implementation of a new policy mandated that physicians pursuing specialty certification after a 2-year postgraduate program avail training at hospitals designated by the Japanese Medical Specialty Board [24]. Although this new and standardized specialty training system increased the number of trainees by 20%, most remained in prefectures that were densely populated, reported lower than the national aging rate of 27%, or had a high doctor density (≧250 doctors per 100,000 people), further worsening the urban-rural inequality in physician distribution [24].

Traditionally, NPPP at the municipality or county level has been used globally as an index of the medical workforce [25]. However, one of the critical shortcomings of NPPP is that it assumes that patients do not seek healthcare outside their municipality of residence [21]. Moreover, as noted in earlier reports, NPPP may have limited applicability in Japan, where a large proportion of municipalities are too small to maintain multi-specialty medical facilities [21]. In addition, although Japan had the lowest difference in the density of physicians between urban and rural communities among OECD countries in 2016 [26], a longitudinal study reported that the NPPP could not detect the shortage of physicians in rural areas [20].

In 2019, MHLW implemented an intervention policy to correct the geographical inequality in physician distribution at a prefectural level using a new policy index called the Physician Uneven Distributed Index (PUDI) [27,28]. The PUDI extends NPPP to include the following three aspects [28]: First, the PUDI includes age- and sex-adjusted consultation rates to account for the differences in healthcare demand due to the changing population structure. Second, the PUDI incorporates the influx and outflux of the population between daytime and nighttime to reflect the healthcare demand more accurately. Third, the PUDI encompasses the sex and age structure of physicians to address their performance. Additionally, MHLW plans to designate some prefectures as physician-saturated prefectures, regulate the number of doctors at the prefectural level, and restrict doctors’ movement to normalize the maldistribution [27,28].

To estimate the relation between the PUDI and healthcare outcomes, we have investigated the association between geographical inequality in physician distribution at a prefectural level measured by the PUDI in 2018 and the CeVD death rate.

## 2. Materials and Methods

### 2.1. Definitions

Physicians Uneven Distribution Index (PUDI), a relatively new index in Japan, was developed to assess the maldistribution of physicians according to the medical demands and supply at the prefectural level. A high PUDI score indicated a high medical supply relative to the medical demand of the area. The formula to calculate PUDI [29] provided by MHLW [28] is as follows:

PUDI = Standardized number of physicians^$^ ÷ (population in the districts ÷ 100,000 × standardized consultation rate of the district^#^)

^*$*^ *Standardized number of physicians = Total number of doctors stratified by gender and 10-year interval age group × (working time for each sex and age group* *[30]* *÷ national mean of working time)* *[31]*

^*#*^ *Standardized consultation rate of the district = Expected consultation rate for the district ÷ national mean of expected consultation rate* ^*§*^ *[32]*

^§^N*ational mean of expected consultation rate = Σ (National mean of age and sex-adjusted consultation rate × by gender and 10-year interval age group) ÷ population of the district)* [33]

### 2.2. Data Source

PUDI data were extracted from the 4th Interim Report of MHLW’s Doctor Supply and Demand Subcommittee [28]. The 2018 NPPP was available in the National Physician Census conducted biennially by the MHLW [19]. The age-adjusted death rate of CeVD data was collected from Vital Statistics every five years from 1998 to 2018 [6]. Population data were collected from the National Basic Resident Register in 2018 [34]. Annual mean income data were collected from the Basic Survey of Wage Structure in 2018 [35]. Crude prevalence of hypertension (based on self-reported health questionnaire that covered the whole household administered nationwide) data were obtained from the Comprehensive Survey of Living Condition in 2016 [36]. A survey for the prevalence of hypertension was not conducted in 2018, and the nearest data available are from 2016. Kumamoto prefecture could not perform the survey in 2016 because of the damage from the Kumamoto earthquake of April 2016.

### 2.3. Outcome Measure

The primary outcome is the age-adjusted CeVD death rate at the prefectural level. The death rate is the number of deaths occurring among the population of a given geographical area (each prefecture in this study) during a given year, per 1000 mid-year total population of the given geographical area during the same year [37]. The age-adjusted death rate is calculated using the 1985 standard population weights.

### 2.4. Other Covariates

We considered population density [38] and the mean annual income [39] as proxy indicators of urbanization [40], and the prevalence of hypertension [41] as a potential confounder for the association between PUDI and the CeVD death rate. The degree of urbanization could affect the physicians’ preference to choose working and living locations [42] and, therefore, the level of CeVD care. Prevalence of hypertension is a well-established cause of CeVD [41] and is associated with many other shared risk factors (such as high salt intake, low-level daily activity, high prevalence of smoking, and low ambient temperature), common in northern parts of Japan where the PUDI is also generally low. We calculated the prevalence of hypertension by dividing the number of patients diagnosed with hypertension in outpatient and inpatient in each prefecture with the prefecture population.

### 2.5. Statistical Analysis

Prefectures were divided into quartiles according to the PUDI score. We calculated mean and standard deviation (SD) for continuous variables. Furthermore, we developed a prefectural level map of PUDI and CeVD death rates to describe the unique geographical distribution in Japan. We also created time-series graphs of CeVD death rates for each prefecture from 1995 to 2018. We assessed the trend of disparity of CeVD death rates using the Gini coefficient. All CeVD death rates were standardized using the 1985′s population model. For the primary analysis, multivariable regression analysis was used to assess the coefficient with PUDI as an independent variable and the CeVD death rate as the dependent variable. We independently included three covariates (population density, mean annual income, prevalence of hypertension) into the regression model. We also performed the same sets of multivariate regression analysis after replacing PUDI with NPPP as an independent variable. For sensitivity analysis, we performed the same analysis after replacing CeVD death rates with cerebral hemorrhage death rate and cerebral infarction death rate, respectively. The terminology of CeVD includes cerebral hemorrhage, cerebral infarction, and other types of cerebrovascular disease in Japanese statistics. We evaluated the linearity of each variable using restricted cubic splines with three knots (two degrees of freedom) [43]. The variance of inflation factor for all variables in the regression models was less than 2.6, which is below the value which is frequently cited as an indicator of multicollinearity [44]. A two-sided *p*-value < 0.05 was considered statistically significant. All analyses were performed using Stata version 16.1 (Stata Corp., College Station, TX, USA).

## 3. Results

**Figure 1:**
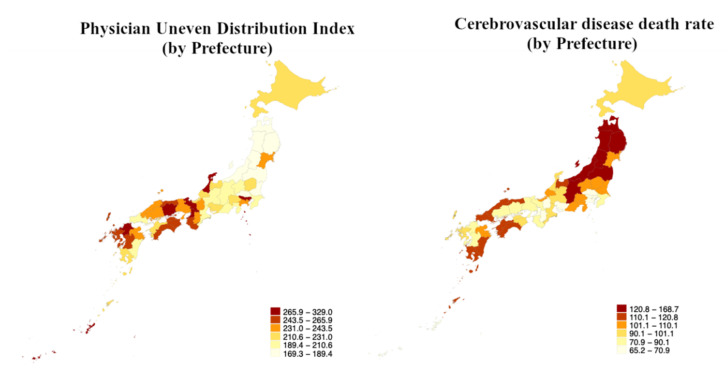
Geographical distribution of Physician Uneven Distribution Index (PUDI) and Cerebrovascular disease (CeVD) death rate in Japan. Northern Honshu has low PUDI and high CeVD death rates.

The geographical distribution of the PUDI and the CeVD death rate among the 47 prefectures of Japan shows that Northern Honshu has a low PUDI and high CeVD death rate (Figure 1). The only exception was Miyagi prefecture, the most populated prefecture in Northern Honshu, which reported high PUDI and low CeVD death rates.

**Figure 2:**
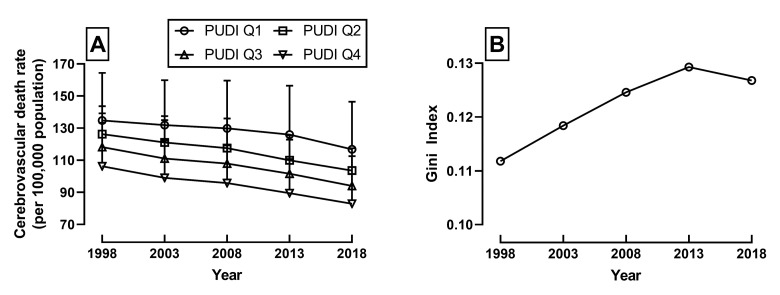
(**A**) CeVD death rate among the 47 prefectures of Japan divided into quartiles by Physician Uneven Distribution Index (PUDI) (*p*-value for trend: Q1= 0.1266, Q2= 0.0004, Q3= 0.0046, and Q4 = 0.0093) and (**B**) increasing disparity of CeVD death rate indicated by increasing Gini index between 1998 and 2018.

Consistent with global trends, the death rate due to CeVD has declined in Japan over the past two decades (Figure 2A). The declining trend of the CeVD death rate was parallel for each quartile by the PUDI. The mean and SD of the CeVD death rate (per 100,000 population) in 1998, 2003, 2008, 2013, and 2018 were 108.4 (22.5), 123.3 (24.8), 117.5 (24.7), 114.5 (25.3), 108.3 (25.0), and 100.8 (23.1), respectively. However, the nationwide disparity in CeVD death rates, as indicated by the Gini index, increased over the 20 years (Figure 2B).

Table 1 shows the characteristics by the quartile of the PUDI. Q1 refers to the lowest, and Q4 refers to the highest group of the PUDI. The CeVD death rate decreased as the PUDI increased, while the population density and the mean annual income increased consistently.

In our main multivariable regression analysis, PUDI was independently associated with CeVD death rates after adjusting for the three covariates (population density, mean annual income, and prevalence of hypertension). The coefficient of the PUDI was −0.34 (95%CI: −0.49–−0.19) before adjusting for covariates and was −0.19 (95%CI: −0.30–−0.07) after adjusting. The adjusted R squared of the main analysis was 0.71 in the final model (Table 2). On the other hand, the same sets of multivariable regression models showed that NPPP was not associated with the CeVD death rate before and after adjusting for covariates. The adjusted R squared of the analysis for NPPP was 0.68 in the final model (Table 3).

The sensitivity analysis by two subset outcomes confirmed the robustness of the association between the PUDI and the CeVD death rate. The PUDI was independently associated with the cerebral hemorrhage death rate and the cerebral infarction death rate after adjusting for the covariates in the same model as the main analysis (Table 4). The coefficient of the PUDI for the cerebral hemorrhage death rate was −0.11 (95%CI: −0.18–−0.03), and the coefficient of the PUDI for the cerebral infarction death rate was −0.06 (95%CI: −0.11–−0.02). The adjusted R squared of the model using the cerebral hemorrhage death rate was 0.71, and the model using the cerebral infarction death rate was 0.48.

## 4. Discussion

There are two important findings from our study: First, even though the overall CeVD death rate has improved in Japan for decades, the inequality of the CeVD death rate at a prefecture-level has increased. Second, the PUDI, the recently adopted policy index reflecting the gap between the medical supply and demand for each prefecture, predicts the CeVD death rate. On the other hand, NPPP, a traditional and widely used policy index of physician maldistribution, was not associated with CeVD death rates.

Indices of physician maldistribution based on the absolute number of doctors such as the NPPP have been used as a guide to increase the number of physicians and as a proxy for the quality of healthcare globally. However, as in the case of Japan, this approach has been unsuccessful elsewhere. For instance, the *Programa Mais Médicos* (The More Doctors program)—a Brazilian government initiative to increase physician supply—exacerbated the uneven distribution further because of the allocation of doctors to non-priority areas [45]. Moreover, an earlier analysis of OECD data for 19 countries failed to identify any association between avoidable mortality and overall physician supply [46]. Additionally, there was no association between avoidable mortality and the per capita supply of general practitioners and family physicians, specialists, or nurses [46].

In an attempt to steer away from models based on the headcount of physicians, several countries, notably the United States and Great Britain, have developed multidimensional indices to measure and reduce physician maldistribution. For example, the Health Resources and Services Administration (HRSA) in the United States designates medically underserved areas using the Medically Underserved Index (MUI) encompassing four variables, namely, the ratio of primary medical care physicians per 1,000 population, the infant mortality rate, the percentage of the population with incomes below poverty level, and the percentage of the population aged 65 or over [47]. Furthermore, HRSA designates the Health Professional Shortage Area (HPSA) using the following three variables: population to provider ratio, percent below the federal poverty level, and travel time to the nearest source of care outside the HPSA designated area [47].

Similarly, the Ministry of Housing, Communities and Local Government (MHCLG) in Britain uses seven variables—income; employment; health and disability; education, skills, and training; barriers to housing and services; crime; and living environment—for its Indices of Deprivation (IoD) to designate deprived areas [48]. However, health deprivation contributes only 13.5% in the total score of the IoD covering premature death, morbidity and disability, emergency admission to hospital, the prevalence of mood and anxiety disorders, hospital episodes, and suicidal mortality among adults in a small local area [48]. 

Although multidimensional indices such as the MUI in the US and the IoD in the UK focuses more on the healthcare outcome to designate a medically deprived area, they do not cover the dimension of medical demand. In contrast to NPPP, the MUI, or IoD, the PUDI incorporates the dimensions of medical supply and demand concurrently and is a unique index to evaluate the uneven distribution of physicians. Given the results of our comparison between NPPP and the PUDI, adding these dimensions of medical supply and demand could improve the sensitivity of the index to detect the inequality of specific health outcomes.

Although the PUDI may be ahead of the headcount approach in considering regional medical supply and demand, its efficacy and impact in normalizing the physician maldistribution are not fully known yet. Our study used the CeVD death rate as a surrogate marker of comprehensive healthcare at the prefectural level, but CeVD is not a universal healthcare policy goal with a global consensus. Therefore, even though our results showed the PUDI excelled NPPP in reflecting the disparity of the CeVD death rate at the prefecture-level, we cannot deduce that improving the PUDI will amend the overall disparity of healthcare. Nonetheless, a previous study reported that optimizing the health supply system could improve outcomes for patients with acute ischemic stroke [49].

Moreover, MHLW aims to further develop the PUDI by incorporating the geographical disadvantage of rural areas [28]. However, the extreme variations in rural disadvantages and challenges in defining and designating rural areas remain a crucial hurdle [50]. It is also worth noting that geographic disparities in the distribution of specialist physicians remain an area of concern in Japan [51] as well as in countries such as the US [52] and Canada [53]. Yet, none of the indices used to assess physician distribution, including the PUDI, reflect the regional demand and supply of the medical subspecialty workforce to deal with the varying burden of specific conditions in one region versus another. Consequently, MHLW is considering implementing a subset version of the PUDI to assess the geographical distribution of specialists, especially obstetrics and pediatrics, and set target goals for those specialties [28]. However, developing such target goals without assessing physicians’ human resources and practice–scope–range is unlikely to have a substantial impact.

Developing a reliable index to evaluate the healthcare of each administrative division will serve as an essential guide for a future health policy to optimize the geographical disparity of healthcare. However, at the same time, the index should be based on evidence because it could have an enormous influence on healthcare delivery. Our findings underscored the importance of incorporating both medical supply and demand concepts to evaluate the healthcare outcome. Further studies that evaluate the association between various healthcare outcomes and the uneven physician distribution are required to establish a reliable and practical index.

In all probability, the COVID19 pandemic has further exacerbated both the shortage and maldistribution of physicians. A significant proportion of healthcare workers have been infected, hospitalized, or deceased during the pandemic [54,55]. Physicians and nurses also report disproportionately higher COVID-19-related stress, anxiety, burnout, and insomnia than the general population [56,57]. The detrimental impact of COVID-19 on our medical workforce is expected as we remerge from the pandemic [58], and future policy directives on physician distribution may have to address unanticipated challenges.

### Limitations

This study has several limitations. First, we only focused on the CeVD death rate as a healthcare outcome, and the results cannot extend to other specific healthcare outcomes. Second, as our results were based on an investigation at the prefectural level, discordance at the town, city, and village levels is very likely and expected. Third, this study used cross-sectional data in 2018. Therefore, the causal effect of PUDI on the CeVD death rate cannot be deduced from our results. Finally, because the data on the prevalence of hypertension was not reported during our study year, we utilized 2016 data, the nearest available survey data to 2018.

## 5. Conclusions

Even though the CeVD death rate in Japan has continuously improved over the past four decades, the disparity in the CeVD death rate gradually increased among prefectures. The newly adopted Physician Uneven Distributed Index (PUDI) reflected the gap between medical supply and demand at the prefectural level and was associated with CeVD death rates. In contrast, the number of physicians per 100,000 population (NPPP), the traditional and widely used policy index of the maldistribution of physicians, was not associated with the CeVD death rates in this study. Therefore, incorporating the gap between the medical supply and demand for each region could improve the responsiveness of the physician maldistribution indices for assessing the disparity in healthcare outcomes.

## Figures and Tables

**Table 1:** Baseline characteristics by quartile of Physicians Uneven Distribution Index (PUDI).

	PUDI = Q1 (*n* = 12)	PUDI = Q2 (*n* = 12)	PUDI = Q3 (*n* = 12)	PUDI = Q4 (*n* = 11)
CeVD death rate (n/100,000)	118 (31)	104 (14)	92 (19)	85 (16)
Population density (n/km2)	0.44 (0.57)	0.34 (0.37)	0.61 (1.02)	1.35 (2.17)
Annual mean income (JPY *, ×10,000/year)	271 (24)	279 (20)	284 (27)	288 (39)
Prevalence of hypertension (%)	13.4 (1.8)	12.1 (1.3)	12.3 (1.9)	11.9 (1.4)

Data are presented as mean (standard deviation). * JPY: Japanese Yen.

**Table 2:** The association between Physicians Uneven Distribution Index (PUDI) and CeVD death rate.

	Model 0, β (95%CI)	Model 1 β (95%CI)	Model 2, β (95%CI)	Model 3, β (95%CI)
PUDI	−0.34 *** (−0.49–−0.19)	−0.25 ** (−0.40–−0.10)	−0.24 *** (−0.36–−0.12)	−0.19 ** (−0.30–−0.07)
Population density		−7.11 ** (−11.75–−2.46)	3.44 (−2.09–8.97)	2.13 (−3.02–7.27)
Annual mean income			−0.60 *** (−0.83 –−0.36)	−0.40 ** (−0.66–−0.15)
Prevalence of hypertension				476.4 ** (153.72–799.05)
n	47	47	47	46
Adjusted R-sq	0.29	0.41	0.62	0.69

Covariates: Population, density, annual mean income, Prevalence of hypertension. ** *p* < 0.01, *** *p* < 0.001.

**Table 3:** The association between the number of physicians per 100,000 people (NPPP) and the CeVD death rate.

	Model 0, β (95%CI)	Model 1, β (95%CI)	Model 2, β (95%CI)	Model 3, β (95%CI)
NPPP *	−0.07 (−0.24–0.10)	−0.06 (−0.20–0.09)	−0.12 (−0.24–0.00)	−0.09 (−0.20–0.02)
Population density		−9.95 *** (−14.71–−5.19)	1.85 (−4.31–8.01)	0.68 (−4.79–6.16)
Annual mean income			−0.66 *** (−0.92–−0.39)	−0.40 ** (−0.68–−0.12)
Prevalence of hypertension				597.40 *** (258.86–935.92)
n	47	47	47	46
Adjusted R-sq	−0.01	0.27	0.52	0.64

Covariates: Population, density, annual mean income, Prevalence of hypertension. ** *p* < 0.01, *** *p* < 0.001.

**Table 4:** Sensitivity analysis: The association between cerebral hemorrhage death rate, cerebral infarction death rate, and Physicians Uneven Distribution Index (PUDI).

	Cerebral Hemorrhage CeVD Rate, β (95%CI)	Cerebral Infarction CeVD Rate, β (95%CI)
PUDI	−0.11 ** (−0.18–−0.03)	−0.06 ** (−0.11–0.02)
Population density	1.58 (−1.65–4.81)	0.60 (−1.45–2.65)
Annual mean income	−0.29 ** (−0.45–−0.13)	−0.09 (−0.19–0.02)
Prevalence of hypertension	307.46 ** (104.92–510.01)	135.93 * (7.38–264.48)
n	46	46
Adjusted R-sq	0.71	0.48

Covariates: Population, density, Annual mean income, Prevalence of hypertension, * *p* < 0.05, ** *p* < 0.01.

## Data Availability

All data we used in this study are open data, and most of them are available from https://www.e-stat.go.jp/, an official portal for Japanese Government Statistics.
